# Society Meetings

**Published:** 1894-02

**Authors:** 


### Society Meetings.

ELEVENTH INTERNATIONAL MEDICAL CONGRESS.—Dr. A. Jacobi, Chairman of the American National Committee, has received a letter, dated December 19, 1893, containing the following communications:

American members will pay on the English, French and Italian railways single fares for double journeys, and will obtain a reduction of twenty per cent. on fares for Italian round-trip tickets.

The documents required for their identification will be sent to you in January, and Americans intending to visit the Congress will have to apply to you for them.

Full particulars concerning the journeys will accompany the documents.

Messrs. Thos. Cook & Son, London, Paris, Rome and Naples, should be applied to for accommodation and for tickets for the excursion at Rome, Naples and to Sicily. Such excursions will be arranged at Rome under the guidance of Mr. Forbes, member of several scientific societies and correspondent of the *Times*—for Naples, three days, including Vesuvius, Pompey, Capri, Sorrento, Castellamare, Bajae, etc.—for Sicily, ten days from Naples, including Messina, Taormina, Catania, Girgenti, Siracusa, Palermo, and return to Naples.

The fares for members of the Congress will be considerably reduced and comprise hotel accommodations, carriages, guides, boats, etc.—about 70 francs each for the three days and 285 francs for the ten days.

Full particulars concerning these excursions will be contained in a leaflet to be added to the instructions and documents for the journey.

From former communications the following are herewith quoted: The members' fee is $5.00; that of their wives or adult relations, $2.00 each. Checks or money orders may be sent to Prof. L. Pagliani, Rome, Italy. Credentials have been promised in the near future. When they arrive (none were received last year), they may be too late for many who have started or are about to start. The undersigned, who is not informed of the cause of the delay, proposes to supply, in as official a form as he thinks he is justified in doing, credentials which are expected to be of some practical value. The North German Lloyd has promised to recognize them. It is suggested, besides, that a passport may increase the traveler's facilities.

Only the North German Lloyd (22 Bowling Green) and the Compagnie Générale Transatlantique (3 Bowling Green) have thought fit to grant any reductions to congressists.

The reductions on Italian railways are available from March 1st to April 30th.

---

THE Medical Society of the State of New York will hold its eighty-eighth annual meeting, Tuesday, Wednesday and Thursday, February 6th, 7th and 8th, in the City Hall, at Albany, commencing at 9.15 A. M., Tuesday, and ending at 1 P. M. on Thursday, under the presidency of Dr. Herman Bendell, of Albany. The following is the provisional program :

*Papers.*—Hemorrhagic Serous Effusion of the Pleura, with Report of a Unique Case, William S. Cheesman, M. D., Auburn ; Researches on the Eliminating Power of Diseases, and the Relation between Vaccinia and Enteric Fever, William Finder, M. D., Troy ; Pneumonia of the Aged, John H. Pryor, M. D., Buffalo ; Diagnosis and Nomenclature of Fevers (second paper), Nelson G. Richmond, M. D., Fredonia ; The Therapeutics of Oxygen, Arnold W. Catlin, M. D., Brooklyn ; Simple Methods in the Diagnosis of Nervous Diseases, E. C. Spitzka, M. D., New York.

*Discussion on Diphtheria.*—(Arranged by A. Walter Suiter, M. D.) Pathology—Status Præsens, Thomas E. Satterthwaite, M. D., New York ; Observations on Diagnosis, and Some Sanitary Aspects, A. Walter Suiter, M. D., Herkimer ; Croup and Diphtheria—Unity or Duality, William H. Daly, M. D., Pittsburg, Pa. ; The Comparative Status of Intubation of the Larynx, Joseph O'Dwyer, M. D., New York ; Complicated Intubation of the Larynx, William Hailes, M. D., Albany ; The Local Treatment, Abraham Jacobi, M. D., New York ; The General Treatment, Edward F. Brush, M. D., Mount Vernon ; The Use of Tartar Emetic in Diphtheria, H. DeV. Pratt, M. D., Elmira.

*Papers.*—Treatment of Depressions in Skull of the New-born, David D. Jennings, M. D., New York ; Immediate Trachelorrhaphy,

Henry C. Coe, M. D., New York; Lympho-adenoma of the Uterus, H. J. Boldt, M. D., New York; Senile Endometritis, A. J. C. Skene, M. D., New York; Treatment of Endometritis, Herman E. Hayd, M. D., Buffalo; Nine Years' Experience with Alexander's Operation for Shortening the Round Ligaments of the Uterus, Paul F. Mundè, New York; Pelvic Abscess, Walter B. Chase, M. D., Brooklyn; A Case of Hysterectomy for Retention of the Menses, William Gardner, M. D., Montreal.

*Discussion.*—(Arranged by Andrew F. Currier, M. D.) Topic, Menstruation and its Abnormalities. Introduction and Normal Function, Andrew F. Currier, M. D., New York; Dysmenorrhea, Its Causes and its Treatment, Howard Kelly, M. D., Baltimore, Md.; Profuse Menstruation, Charles P. Noble, M. D., Philadelphia, Pa.; Scanty Menstruation, Franklin Townsend, Jr., M. D., Albany; Irregular Menstruation, Charles A. L. Reed, M. D., Cincinnati, O., and E. W. Cushing, M. D., Boston, Mass.; Menopause, Natural and Artificial, Arthur W. Johnstone, M. D., Cincinnati, O.

*Papers.*—Urethral Caruncles, Edward M. Liell, M. D., New York; The Physical Causes of Sexual Debility in the Male, as Distinguished from the Psychical Causes, F. R. Sturgis, M. D., New York; The Surgical Treatment of the Prostate Gland, Seneca D. Powell, M. D., New York; The Fable of the Egg, William S. Ely, M. D., Rochester; Artificial Immunity, Henry R. Hopkins, M. D., Buffalo; Clinical Notes on Psoriasis, with Especial Reference to its Prognosis and Treatment, L. Duncan Bulkley, M. D., New York; Spinal Supports and Braces, the Indications for Their Use, History and Modern Perfection (to be illustrated with forty lantern slides), A. M. Phelps, M. D., New York; History and Pathology of the Spinal Cord (illustrated with lantern slides), William C. Krauss, M., D., Buffalo.

*Discussion on Abdominal Surgery.*—(Arranged by A. Vander Veer, M. D.) Disputed Points in the Treatment of Pelvic Surgery, Joseph Price, M. D., Philadelphia, Pa.; Influences Affecting the Results of Abdominal Operations, J. F. W. Ross, M. D., Toronto, Canada; Hemorrhage After Abdominal Section, Its Place in Statistics, A. H. Buckmaster, M. D., New York; Cysts of the Epigastrium, Dudley P. Allen, M. D., Cleveland, O.; The Technique of the Abdominal Incision, Methods of its Closure and its Subsequent Management, William Warren Potter, M. D., Buffalo; Operative Procedure for the Relief of Obstruction of the Common Duct, W. E. B. Davis, M. D., Birmingham, Ala.; Two Cholecystotomies for Gall-Stones with Recovery, with Remarks on Operative Methods Based upon Five Cases, William Wotkyns Seymour, M. D., Troy; Gall-Stones, the Exciting Cause of Malignant Disease, Rufus B. Hall, M. D., Cincinnati, O.; Appendicitis, Charles McBurney, M. D., New York; An Analysis of 150 Personally Observed Cases of Appendicitis, George Ryerson Fowler, M. D., Brooklyn; A Conservative View of the Treatment of Appendicitis, William S. Tremaine, M. D., Buffalo; Some Observations Relative to the Treatment of Suppurative Appendicitis, with Report of Cases, Willis G. Macdonald, M. D., Albany; Palpation of the Vermiform Appendix, G. M. Edebohles, M. D., New York; The Inch and a Half Incision, and Week and a Half Confinement in Appendicitis, Robert T. Morris, M. D., New York; Report of a Case of Post-Peritoneal Abscess from Duodenal Ulcer, with Presentation of Specimen, L. S. Pilcher, M. D., Brooklyn; Intestinal Perforation in Strangulated Hernia, William B. DeGarmo, M. D., New York; Remarks on the After-Treatment of Abdominal Section, Carlton C. Frederick, M. D., Buffalo; The Unexpected as Sometimes Oberved in Abdominal Surgery, A. Vander Veer, M. D., Albany.

*Papers.*—Recent Methods of Gastrostomy for Stricture of the Esophagus, Willy Meyer, M. D., New York; The Influence of Physiological Rest on Prolapse of the Rectum, Joseph D. Bryant, M. D., New York; A Contribution to the Subject of Excision of the Larynx, Charles A. Powers, M. D., New York; Observations on 118 Cases of Cancer of the Breast, with Especial Reference to its Radical Cure by Operation, William T. Bull, M. D., New York; The Treatment of Hernia (supplement to paper read last year), Alexander Dallas, M. D., New York; Some Cases of Brain Surgery, Herman Mynter, M. D., Buffalo; The Needlessness of a Mydriatic in Adjusting Glasses to the Eye, D. B. St. John Roosa, M. D., New York; The Action of Scopolamine on the Eye, Thomas R. Pooley, M. D., New York; The Treatment of Nasal Hemorrhage, John O. Roe, M. D., Rochester; Report of a Case of Injury to Cauda Equina, Hermon C. Gordinier, M. D., Troy; The Treatment and Prevention of Epilepsy in the Young, Graeme M. Hammond, M. D., New York; The Practical Workings of the Law for the Care of the Insane, Carlos F. Macdonald, M. D., New York; Lunatics in Public Places, Wallace J. Herriman, M. D., Rochester; The Subfrontal Gyre (Brocà's Convolution) in Man and Apes, Burt G. Wilder, M. D., Ithaca; Acromegaly, Floyd S. Crego, M. D., Buffalo; Report of a Case of Acromegaly, with the Exhibition of the Subject, Frederick Remington, M. D., Rochester; Uremic Hemiplegia, Reynold W. Wilcox, M. D., New York; Glycosuria, W. B. Vanderpoel, M. D., New York.

